# Decreased serum betatrophin may correlate with the improvement of obstructive sleep apnea after Roux-en-Y Gastric Bypass surgery

**DOI:** 10.1038/s41598-021-81379-1

**Published:** 2021-01-19

**Authors:** Zhiyuan Song, Kaifeng Guo, Weijun Huang, Huajun Xu, Yupu Liu, Jian Guan, Shankai Yin, Haoyong Yu, Hongliang Yi, Jianyin Zou

**Affiliations:** 1 Department of Otolaryngology-Head and Neck Surgery, Shanghai Jiao Tong University Affiliated Sixth People’s Hospital, 600 Yishan Road, Shanghai, 200233 China; 2 Shanghai Key Laboratory of Sleep Disordered Breathing, Shanghai, 200233 China; 3 Otolaryngology Institute of Shanghai Jiao Tong University, Shanghai, 200233 China; 4 Department of Endocrinology and Metabolism, Shanghai Clinical Center for Diabetes, Shanghai Diabetes Institute, Shanghai Key Laboratory of Diabetes Mellitus, Shanghai Key Clinical Center for Metabolic Disease, Shanghai Jiao Tong University Affiliated Sixth People’s Hospital, 600 Yishan Road, Shanghai, 200233 China; 5 Department of Endocrinology and Metabolism, Minhang Hospital, Fudan University, Shanghai, 201199 China; 6 Minhang Branch, Zhongshan Hospital, Fudan University, Shanghai, 201199 China; 7 Central Hospital of Minhang District, Shanghai, 201199 China

**Keywords:** Endocrinology, Risk factors

## Abstract

Obesity is strongly correlated with obstructive sleep apnea (OSA), and bariatric surgery can effectively treat obesity and alleviate OSA. However, the contributing factors are still unclear. We aimed to explore the relationship between betatrophin and OSA in patients undergoing Roux-en-Y gastric bypass (RYGB) surgery. Our study consisted of thirty-seven individuals with OSA and type 2 diabetes (16 males, 21 females) undergoing RYGB surgery. The polysomnography test, anthropometric results, serum betatrophin, and abdominal magnetic resonance images were evaluated both before and 1 year after RYGB surgery. Factors that may correlate with the alleviation of OSA were investigated. In our study, RYGB surgery significantly decreased apnea hypopnea index (AHI) and serum betatrophin concentration (*p* < 0.001). The abdominal visceral fat area, subcutaneous fat area and HOMA-IR were also significantly decreased (*p* < 0.001). The preoperative AHI, postoperative AHI and the change in AHI were significantly correlated with the preoperative betatrophin, postoperative betatrophin and the change in betatrophin, respectively (*p* < 0.05). These correlations were still significant after adjustment for other risk factors. The change in betatrophin was also independently associated with the change in minimum oxygen saturation (*p* < 0.001). Our data might indicate that serum betatrophin was significantly independently correlated with the improvement of OSA after bariatric surgery.

## Introduction

Obstructive sleep apnea (OSA) is a common disease characterized by recurrent episodes of partial or complete airway obstruction resulting in hypoxemia, hypercapnia, and respiratory arousal^1^. The prevalence rate of OSA is ~ 4% among various middle-aged male populations, and represents a health risk through direct and indirect mechanisms^2^. Moreover, it is associated with a fivefold increased risk of metabolic syndrome, such as abnormal lipid metabolism and insulin resistance, in comparison to healthy individuals^3^. Obesity is considered the strongest risk factor for OSA, because obesity-related systemic inflammatory mediators, localization of excess adipose tissue, reduced chest wall compliance, disturbances in the relationship between respiratory drive and load compensation, reductions in functional residual capacity and hormonal changes may have adverse effect in pharyngeal neural and mechanical control, and weight loss could significantly alleviate the severity of OSA for these possible mechanisms^4^. Bariatric surgery, one of the most common and effective treatments for obesity, has also been confirmed to be effective to improve OSA^5^.

However, the underlying factor by which weight loss contributes to alleviation of OSA is unclear. Previous studies have indicated that greater abdominal fat accumulation was associated with more serious OSA^6^, and changes in visceral adipose tissue volume showed a strong relationship with improvement of OSA^7^. However, reduced regional fat distribution can only partially explain the alleviation of OSA^8^. Weight loss may also influence OSA according to its metabolic consequences^9,10^. Recent evidence indicated that there are changes in inflammatory biomarkers and adipokines after bariatric surgery^11,12^, but the associations between these changes and alleviation of OSA have not been studied in detail. Betatrophin, known as angiopoietin-like protein 8 or lipasin, is a hormone found in the liver and adipose tissue, and is a potent regulator of lipid metabolism^13^. Our previous study indicated a significant decrease in serum betatrophin after Roux-en-Y gastric bypass (RYGB) surgery^14^. Recently, two studies reported a higher serum betatrophin concentration in the OSA group than in the control group^15,16^. However, the association between serum betatrophin and OSA have not been well evaluated, especially in people with weight loss. This study was performed to evaluate the changes in serum betatrophin in obese patients with OSA after RYGB surgery, and to examine any association between serum betatrophin and OSA.

## Methods

### Participants and measurements

Thirty-seven consecutive Chinese obese patients with OSA and type 2 diabetes (T2D), who had received RYGB surgery at our hospital, were recruited to this longitudinal retrospective study. Diagnosis of T2D was according to the American Diabetes Association (ADA) diagnostic standard of 2007. RYGB is performed to treat obesity and T2D and is appropriate for Chinese T2D patients with a BMI of 25–35 kg/m^2^^17^. OSA was defined according to the American Academic Sleep Medicine (AASM) criteria^18^. All participants were aged 20–70 years. Patients with psychiatric disturbances and those undergoing continuous positive airway pressure treatment, systemic steroid treatment or hormone-replacement therapy were excluded. All participants provided written informed consent before inclusion in the study. This study was approved by the Ethics Committee of the Shanghai Jiao Tong University Affiliated Sixth People's Hospital and complied with the Declaration of Helsinki.

Before and 1 year after RYGB surgery, all subjects were asked to undergo an overnight polysomnography (PSG) test in the sleep center at our hospital. During the sleep center visit, all participants were asked to complete the Epworth Sleepiness Scale (ESS) before the overnight PSG test. Fasting blood samples were taken the next morning in the fasting state and immediately after the PSG test. Body habitus, including weight, height, neck circumference (NC), waist circumference (WC), and hip circumference (HC), was measured using standard anthropometric methods. Blood samples were collected to measure fasting plasms glucose (FPG), fasting insulin, lipids, and betatrophin levels. Both the surgical procedure and the measurements of blood samples were as described previously^14^. Serum betatrophin was determined using commercially available enzyme-linked immunosorbent assays (ELISAs; SK00528-02, Aviscera Bioscience Inc., Santa Clara, CA, USA) according to the manufacturer’s instructions^19^. The homeostasis model assessment of insulin resistance (HOMA-IR) was used as a parameter to evaluate the degree of insulin resistance. Subcutaneous fat area (SFA), visceral fat area (VFA), and total fat area (TFA) were assessed in each participant at baseline and 1 year after RYGB using a magnetic resonance imaging (MRI) system (Achieva 3.0-T; Philips Medical Systems, Eindhoven, The Netherlands) with standard array coils, with the subject in the supine position.

### Polysomnography test

A laboratory-based PSG (Alice 4; Respironics Inc., Pittsburgh, PA) was used to diagnose OSA. PSG records were evaluated manually according to standard criteria by a single skilled technician^20^. The apnea hypopnea index (AHI) was defined as the number of apnea and hypopnea events per hour during sleep. The parameters of mean oxygen saturation (SaO_2_), minimum SaO_2_ and the percentage of time spent at SaO_2_ < 90% (CT90%) were also included in the data analysis. Patients with AHI < 5 events/h before RYGB surgery were excluded from the follow-up study.

### Statistical analysis

Continuous variables are presented as means ± standard deviation, except for skewed variables, which are presented as the mean (95% confidence interval, CI). Categorical variables are expressed as percentages. Differences between baseline and postoperative characteristics of the participants were examined using the paired Student’s *t* test, Wilcoxon’s signed-rank test, Kruskal–Wallis test, or χ^2^ test, as appropriate. Correlations of the various variables and PSG parameters were analyzed using Spearman’s correlation test or Pearson correlation test. Parameters that may influence the effect of RYGB surgery on OSA were evaluated by partial correlation analysis. We considered *p* < 0.05 to indicate statistical significance for a two-sided test. All statistical analyses were performed using SPSS software (version 19.0 for Windows; SPSS Inc., Chicago, IL).

## Results

### Clinical characteristics of the subjects at baseline and 1 year after RYGB surgery

**Figure 1:**
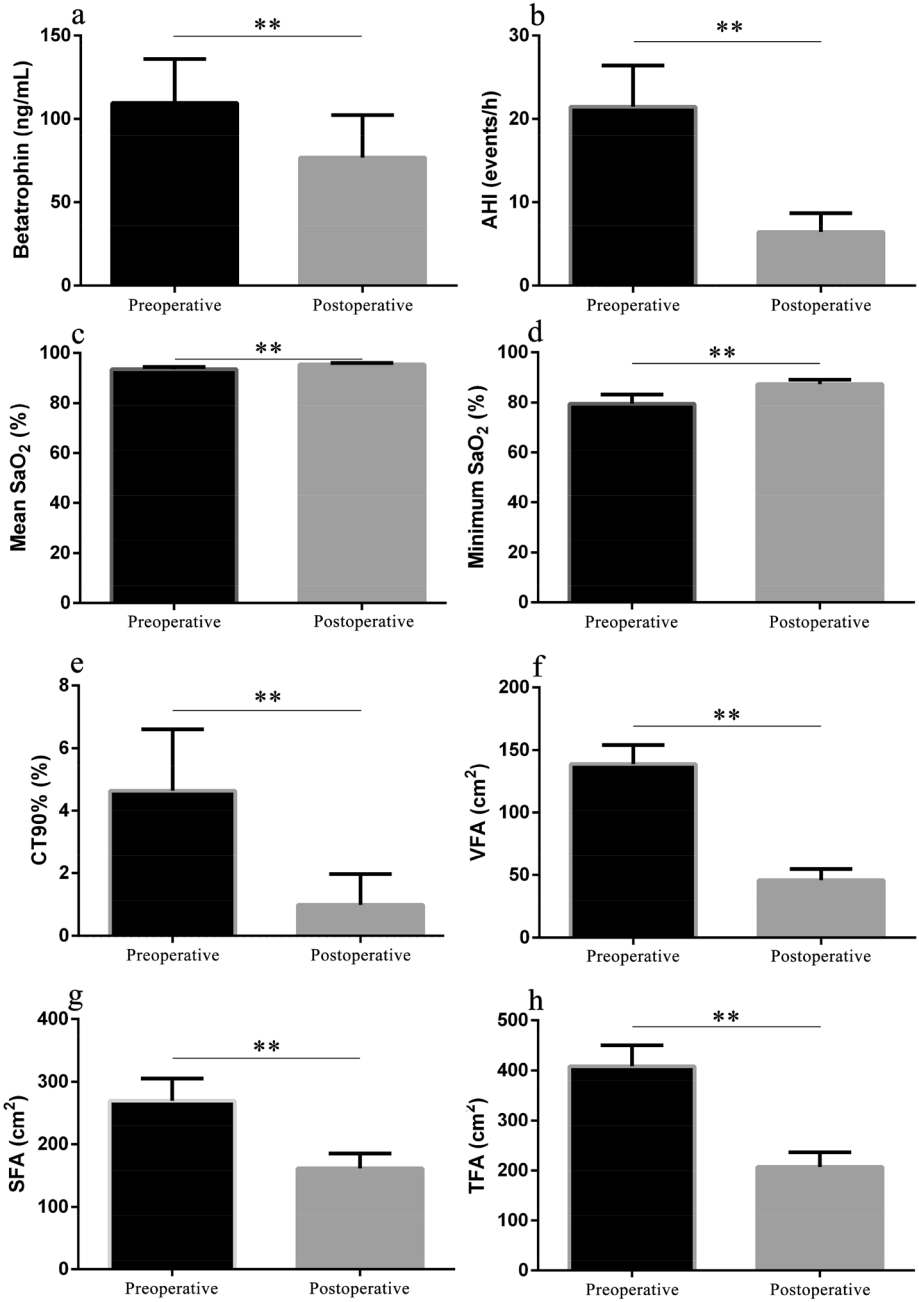
Comparisons of parameters of betatrophin (**a**), AHI (**b**), mean SaO_2_ (**c**), minimum SaO_2_ (**d**), CT90% (**e**), VFA (**f**), SFA (**g**) and TFA (**h**) between baseline and one year after RYGB. **p < 0.001. Data were shown as mean (95% confidence interval). *RYGB* Roux-en-Y gastric bypass, *AHI* apnea hypopnea index, *SaO*_*2*_ oxygen saturation, *CT90%* percentage of time spent at SaO_2_ < 90%, *VFA* visceral fat area, *SFA* subcutaneous fat area, *TFA* total fat area.

The demographics and clinical characteristics of the subjects are shown in Table 1. Of the total population of 37 patients, 16 were male and 21 were female. The mean age of the study population was 49.3 ± 10.6 years. Compared with baseline values, BMI, NC, WC, HC, FPG, fasting insulin, HOMA-IR, and the ESS score were all significantly decreased 1 year later (all *p* < 0.001). The mean BMI dropped from 31.0 ± 3.4 to 24.0 ± 2.2 kg/m^2^. The mean total weight loss was 17.9 ± 6.8 kg. Significant changes were also observed in lipid profiles, such as total cholesterol, triglyceride, low-density lipoprotein, and high-density lipoprotein (all *p* < 0.001). As shown in Fig. 1, serum betatrophin level decreased significantly from 109.55 ng/mL (95% CI 83.26–135.84) to 76.71 ng/mL (95% CI 51.09–102.33) (*p* < 0.001). The parameters of abdominal fat accumulation were also significantly decreased at follow-up; the baseline values of TFA, SFA, and VFA were 408.6 cm^2^ (95% CI 366.9–450.3), 269.7 cm^2^ (95% CI 234.4–305.0), and 138.9 ± 45.3 cm^2^, while those at 1 year later had decreased to 207.4 cm^2^ (95% CI 178.3–236.5), 161.4 cm^2^ (95% CI 137.7–185.2), and 45.9 ± 26.6 cm^2^, respectively (all *p* < 0.001). PSG indicators showed that the AHI changed significantly from 21.5 events/h (95% CI 16.5–26.4) to 6.4 events/h (95% CI 4.2–8.7) (*p* < 0.001). The minimum SaO_2_ was 87% (95% CI 86–89%) after surgery compared to 80% (95% CI 76–83%) at baseline (*p* < 0.001).

**Table 1:** Clinical characteristics of the patients at baseline and one year after RYGB.

Indicators	Preoperative	Postoperative
BMI (kg/m^2^)	31.0 ± 3.4	24.0 ± 2.2**
NC (cm)	39.4 (38.4–40.5)	35.6 (34.4–36.8)**
WC (cm)	103.3 (99.6–107.0)	84.8 (82.4–87.3)**
HC (cm)	106.5 (103.4–109.7)	94.4 (92.5–96.3)**
WC/HC	0.97 ± 0.05	0.90 ± 0.05**
FPG (mmol/L)	9.33 (8.01–10.65)	5.91 (5.49–6.32)**
Fasting insulin (mU/L)	20.85 (13.79–27.91)	7.23 (4.44–10.03)**
HOMA-IR	9.03 (5.34–12.72)	1.94 (1.13–2.76)**
ESS	7.2 (5.8–8.7)	3.4 (2.4–4.4)**
Total cholesterol (mmol/L)	5.10 (4.81–5.40)	4.20 (3.93–4.47)**
Triglyceride (mmol/L)	2.79 (1.69–3.89)	1.02 (0.88–1.15)**
High-density lipoprotein (mmol/L)	0.97 (0.91–1.04)	1.21 (1.12–1.30)**
Low-density lipoprotein (mmol/L)	2.94 ± 0.89	2.50 ± 0.68**

Data are expressed as the mean ± SD or mean with 95% confidence interval.

*RYGB* Roux-en-Y gastric bypass, *BMI* body mass index, *NC* neck circumference, *WC* waist circumference, *HC* hip circumference, *FPG* fasting plasma glucose, *HOMA-IR* homeostasis model assessment of insulin resistance, *ESS* Epworth sleepiness scale.

**p < 0.001.

### Factors associated with the alleviation of OSA

**Figure 2:**
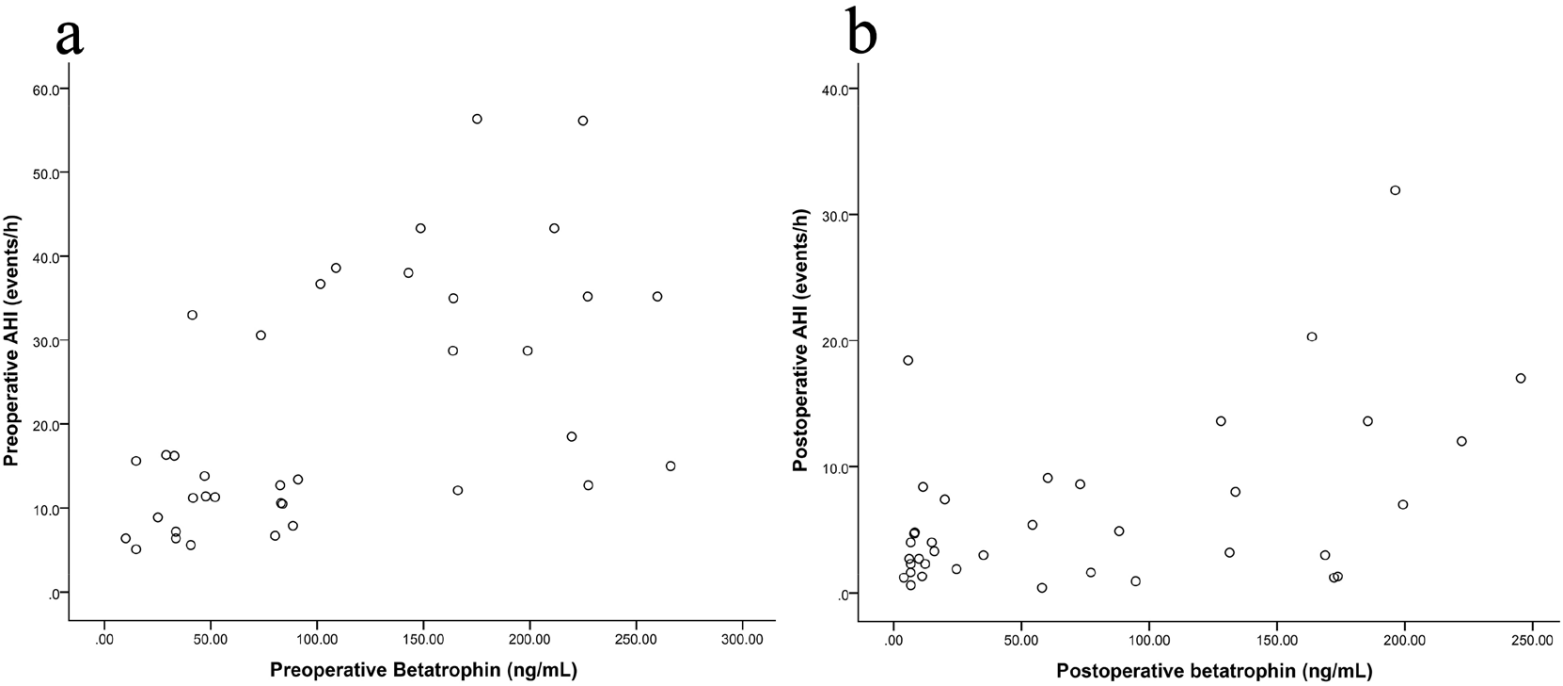
(**a**) A Bland–Altman plot of the preoperative AHI versus the preoperative betatrophin. (**b**) A Bland–Altman plot of the postoperative AHI versus the postoperative betatrophin. *AHI* apnea hypopnea index.

Spearman correlation analysis indicated that preoperative AHI was significantly correlated with preoperative betatrophin (*r* = 0.603, *p* < 0.001, Fig. 2a), ESS score (*r* = 0.636, *p* < 0.001), BMI (*r* = 0.431, *p* = 0.008) and NC (*r* = 0.382, *p* = 0.02) at baseline. When adjusted for the other factors, preoperative AHI was significantly independently correlated with preoperative betatrophin (*r* = 0.439, *p* = 0.009). Preoperative minimum SaO_2_ was significantly correlated with preoperative betatrophin (*r* = − 0.458, *p* = 0.004,), ESS score (*r* = − 0.476, *p* = 0.003), VFA (*r* = − 0.434, *p* = 0.007), BMI (*r* = − 0.397, *p* = 0.015), NC (*r* = − 0.439, *p* = 0.007), WC (*r* = − 0.360, *p* = 0.029) and HC (*r* = − 0.365, *p* = 0.026) at baseline. However, when adjusted for the other factors, preoperative minimum SaO_2_ was not significantly correlated with preoperative betatrophin (*p* > 0.05).

One year after bariatric surgery, 23 patients with OSA were cured, while the other 14 patients had residual OSA. Parameters that were significantly different between these two groups were shown in Table 2. Postoperative AHI was significantly correlated with postoperative betatrophin (*r* = 0.356, *p* = 0.031, Fig. 2b), ESS score (*r* = 0.533, *p* = 0.001), and waist–hip ratio (*r* = 0.346, *p* = 0.036). When adjusted for the other factors, postoperative AHI was significantly independently correlated with postoperative betatrophin (*r* = 0.431, *p* = 0.01).

**Table 2:** Differences between normal group and OSA group after bariatric surgery.

Indicators	Normal group	OSA group
Age (years)	48.6 ± 11.4	50.5 ± 9.5
BMI (kg/m^2^)	23.8 ± 2.1	24.4 ± 2.5
NC (cm)	34.9 (33.7–36.1)	36.7 ± 4.8
WC (cm)	83.7 (80.7–86.8)	86.6 (82.1–91.2)
HC (cm)	93.9 (91.6–96.2)	95.3 (91.7–98.9)
WC/HC	0.89 ± 0.04	0.91 ± 0.06
FPG (mmol/L)	5.61 (5.05–6.18)	6.39 (5.81–6.97)*
Fasting insulin (mU/L)	5.55 (3.90–7.19)	10.0 (2.77–17.23)
HOMA-IR	1.41 (0.85–1.97)	2.82 (0.78–4.86)*
ESS	2.2 ± 2.1	5.3 ± 3.3**
Total cholesterol (mmol/L)	4.08 ± 0.84	4.39 ± 0.74
Triglyceride (mmol/L)	0.89 ± 0.33	1.22 ± 0.43*
High-density lipoprotein (mmol/L)	1.23 ± 0.27	1.19 ± 0.26
Low-density lipoprotein (mmol/L)	2.49 (2.23–2.75)	2.53 (2.05–3.00)
VFA (cm^2^)	42.0 ± 23.3	52.4 ± 31.0
SFA (cm^2^)	157.6 (128.9–186.4)	167.7 (121.3–214.1)
TFA (cm^2^)	199.6 (164.7–234.6)	220.1 (163.1–277.1)
Betatrophin (ng/mL)	49.57 (23.80–75.35)	121.3 (73.4–169.2)**
AHI (events/h)	2.5 ± 1.4	12.9 ± 7.1**
Mean SaO_2_ (%)	95.9 ± 1.3	94.7 ± 1.9*
Minimum SaO_2_ (%)	89.5 ± 2.3	83.9 ± 6.5**
CT90% (%)	0.1 (0–0.3)	2.3 (0.3–4.5)*

Data are expressed as the mean ± SD or mean with 95% confidence interval.

*OSA* obstructive sleep apnea, *BMI* body mass index, *NC* neck circumference, *WC* waist circumference, *HC* hip circumference, *FPG* fasting plasma glucose, *HOMA-IR* homeostasis model assessment of insulin resistance, *ESS* Epworth sleepiness scale, *VFA* visceral fat area, *SFA* subcutaneous fat area, *TFA* total fat area, *AHI* apnea hypopnea index, *SaO*_*2*_ oxygen saturation, *CT90%* percentage of time spent at SaO_2_ < 90%.

*p < 0.05.

**p < 0.01.

Spearman correlation analysis also indicated the change in AHI was significantly associated with the change in betatrophin (*r* = 0.421, *p* = 0.009, Table 3) and ESS score (*r* = 0.540, *p* < 0.001). When adjusted for the change in ESS score, the change in AHI was significantly independently correlated with the change in betatrophin (*r* = 0.515, *p* = 0.001). The change in the minimum SaO_2_ was also significantly associated with changes in betatrophin (*r* = − 0.468, *p* = 0.004), the ESS score (*r* = − 0.388, *p* = 0.018) and HC (*r* = − 0.356, *p* = 0.031). When adjusted for the other factors, the change in betatrophin was independently associated with the change in AHI (*r* = 0.515, *p* = 0.001) and the minimum SaO_2_ (*r* = − 0.579, *p* < 0.001).

**Table 3:** Factors associated with the alleviation of OSA.

Indicators	△AHI	△mean SaO_2_	△minimum SaO_2_	△CT90%
△BMI (kg/m^2^)	0.208	0.061	− 0.030	0.397*
△NC (cm)	0.069	− 0.022	0.217	− 0.192
△WC (cm)	0.134	0.115	− 0.206	0.326*
△HC (cm)	0.224	− 0.071	− 0.356*	0.244
△WC/△HC	− 0.090	0.309	0.104	0.153
△FPG (mmol/L)	− 0.024	− 0.050	0.260	− 0.134
△Fasting insulin (mU/L)	0.012	− 0.074	0.083	0.123
△HOMA-IR	− 0.045	− 0.105	0.226	− 0.034
△ESS	0.540**	− 0.366*	− 0.388*	0.545**
△Total cholesterol (mmol/L)	0.053	− 0.419*	− 0.208	0.356*
△Triglyceride (mmol/L)	0.267	− 0.051	− 0.218	0.124
△High-density lipoprotein (mmol/L)	0.026	0.188	− 0.143	− 0.027
△Low-density lipoprotein (mmol/L)	− 0.052	− 0.304	0.072	0.056
△VFA (cm^2^)	0.241	− 0.076	− 0.225	0.430**
△SFA (cm^2^)	− 0.047	0.093	0.154	0.019
△TFA (cm^2^)	0.096	0.040	0.028	0.222
△Betatrophin (ng/mL)	0.421**	− 0.218	− 0.468**	0.102

*OSA* obstructive sleep apnea, *AHI* apnea hypopnea index, *SaO*_*2*_ oxygen saturation, *CT90%* percentage of time spent at SaO_2_ < 90%, *BMI* body mass index, *NC* neck circumference, *WC* waist circumference, *HC* hip circumference, *FPG* fasting plasma glucose, *HOMA-IR* homeostasis model assessment of insulin resistance, *ESS* Epworth sleepiness scale, *VFA* visceral fat area, *SFA* subcutaneous fat area, *TFA* total fat area.

*p < 0.05.

**p < 0.01.

## Discussion

Bariatric surgery can effectively treat obesity and improve OSA^21^. In this study, betatrophin, physical parameters (BMI, NC, WC, etc.), indices of lipid metabolism (triglyceride, cholesterol, etc.), OSA indictors (AHI, the minimum SaO_2_, etc.), and body fat distribution parameters (SFA, VFA, and TFA) showed significant changes after surgery. The change of betatrophin was found to be independently correlated with the alleviation of OSA. Therefore, adipokines, such as betatrophin, may be associated with the improvement of OSA after RYGB surgery.

Obesity has been widely reported and accepted as the most frequent risk factor for OSA^22,23^. Weight reduction has been demonstrated as an important treatment for OSA, because even a small decrease in BMI can significantly improve the AHI^24^. However, the extent of weight loss did not correlate with OSA improvement^25^, which has also been found in our previous study^26^. Some authors have attributed this to the interaction of anatomic factors^27^ and weight-independent metabolic effects^28^, such as the cytokines, gut hormones and adipokines. However, the current evidence could only partially explain the alleviation of OSA, which means further research are required to reveal the underlying mechanisms of OSA resolution after weight loss.

Betatrophin is a hormone highly enriched in the liver and adipose tissues, and has been shown to be relevant to obesity and glucose/lipid homeostasis^29,30^. Both in vivo and in vitro models, inhibition of betatrophin leads to the phenotype change of adipocytes characterized by increased mitochondria contents, beige adipocytes and mitochondria biogenesis-specific markers^31^. Betatrophin has also been reported to regulate the lipoprotein lipase activity in the heart and skeletal muscles^32^, and regulate the energy homeostasis. Suppressed betatrophin could lead to lipoprotein lipase activation in muscles and triglyceride mobilization to muscles for oxidation and energy supply with greater expression of genes related to beta-oxidation^33,34^, which may enhance upper airway muscle function and result in the alleviation of OSA. The lipoprotein lipase has also been demonstrated to be downregulated by hypoxia^32^. Considering the association between OSA and obesity and dyslipidemia, the potential interaction between OSA and betatrophin is worth exploring. However, there have been few studies regarding the association between betatrophin and OSA. It was not until recently that two studies reported the serum betatrophin concentration was higher in the OSA group than in the control group^15,16^, and this was consistent with our study. Moreover, our study showed a reduction of serum betatrophin after the alleviation of OSA, and the change of betatrophin was significantly correlated with the change of AHI and the change of minimum SaO_2_. Increasing evidence has indicated that serum betatrophin concentration is influenced by serum lipid profile, obesity and T2D. However, when adjusted for the influence factors in our study, the change of betatrophin was still significantly correlated with the change of AHI and the minimum SaO_2_. The results of these previous studies taken together with our findings suggest that betatrophin may be associated with the alleviation of OSA. Although the complex association between betatrophin and OSA could not reveal causal relations, our results might provide supplementary clinical evidence for a new perspective for the study of OSA.

This study also had some limitations. First, it was carried out in subjects with OSA and T2D rather than patients with simple obesity, so the generalizability of the results to other populations is unclear. Second, adipose tissue-related factors and gastrointestinal hormones were not exhaustively measured in this study, and the research evidence between betatrophin and OSA could not reveal underlying mechanism. Moreover, the small sample size, lack of a nonsurgical weight loss group and limited follow-up duration may also limit the generalizability of the results. Therefore, further studies including larger samples are required to further examine the underlying role of betatrophin, as well as other metabolic factors, on the therapeutic effects of RYGB surgery on OSA.

## Conclusions

This is the first study showing significant reduction in the serum betatrophin level after RYGB surgery in obese Chinese patients with T2D. Serum betatrophin may be associated with the improvement of OSA after bariatric surgery. Further prospective studies are required to determine the mechanisms underlying these observations.

## Data Availability

The datasets generated during and/or analysed during the current study are available from the corresponding author on reasonable request.
